# Interleukin 21 (IL-21) regulates chronic allograft vasculopathy (CAV) in murine heart allograft rejection

**DOI:** 10.1371/journal.pone.0225624

**Published:** 2019-11-22

**Authors:** Mithun Khattar, Caitlin E. Baum, Paul Schroder, Joshua D. Breidenbach, Steven T. Haller, Wenhao Chen, Stanislaw Stepkowski

**Affiliations:** 1 Department of Medical Microbiology and Immunology, The University of Toledo College of Medicine and Life Sciences, Toledo, OH, United States of America; 2 Department of Surgery, Duke University Medical Center, Durham, NC, United States of America; 3 Department of Medicine, The University of Toledo College of Medicine and Life Sciences, Toledo, OH, United States of America; Université Paris Descartes, FRANCE

## Abstract

IL-21 is the most recently discovered common gamma-chain cytokine that promotes persistent T-cell responses in chronic infections, autoimmunity and cancer. However, the therapeutic potential of inhibiting the IL-21-BATF signaling axis, particularly in transplant rejection, remains unclear. We used heart transplant models to examine the effects of IL-21 blockade in prevention of chronic cardiac allograft vasculopathy (CAV) using genetic knock-out and therapeutic approaches. Both wild-type C57BL/6 and IL-21-/- strains acutely rejected Balb/c skin grafts and once immunized with this skin graft, rejected Balb/c heart allografts in an accelerated fashion. However, when transplanted with heart grafts from the class-II major histocompatibility complex mutant, B6^bm12^ mice; wild-type recipients developed CAV, while IL-21-/- recipients were protected, even at day 100 post-transplant. Similarly, BATF-/- recipients, lacking the transcription factor BATF responsible for IL-21 production, did not develop CAV in B6-bm12 heart allografts. Strikingly, in a transient treatment protocol, the development of CAV in wild-type recipients of B6-bm12 hearts allografts was blocked by the administration of IL-21 receptor fusion protein (R-Fc). Thus, we demonstrate that CAV is regulated at least in part by IL-21 signaling and its blockade by genetic approaches or therapy with IL-21R-Fc prevents CAV in mice.

## Introduction

While advances in immunosuppressive therapies and donor selection through immunological pre-testing have greatly improved the survival of heart transplants, their long-term survival remains a challenge. Long-term survival is especially hindered by the process of cardiac allograft vasculopathy (CAV), which is a slowly progressing occlusion of the coronary arteries beginning immediately after transplantation[1]. Pathologically, CAV is characterized by concentric intimal smooth muscle hyperplasia through the entire length of the vessel, gradually obstructing its lumen. The process of CAV is also associated with local inflammatory infiltrates such as macrophages and lymphocytes. Sometimes, human heart transplants develop CAV relatively quickly producing fatal graft failure within 1–2 years post-transplant. Other times, CAV develops slowly contributing to the eventual graft failure often years after transplantation[2]. The development of CAV has been linked to both immunologic factors such as anti-endothelial cell antibodies or T-cells and complement activation as well as non-immunologic risk factors such as hypertension[3–5]. Since CAV development in heart transplants is responsible for up to 30% of deaths by 5-years post-transplant, finding an effective therapy should be a priority in heart transplantation research[6].

Recent publications provide new data on the multifactorial role of IL-21 in regulating B-cells and several T-cell subsets such as T follicular helper (Tfh) and T helper 17 (Th17)[7, 8]. Indeed, the signal transducer and activator of transcription 3 (STAT3)-dependent survival of Tfh and Th17 cells distinguish them from STAT5-dependent survival of Th1 and Th2[9, 10]. An elegant study demonstrated that IL-21 was required to control chronic but not acute viral infections in mice[11]. Similarly, the critical role of IL-21 signaling has been well-demonstrated in chronic auto-immune diseases like type-1 diabetes[12]. In transplant recipients, increased IL21 signaling is shown to be associated with reduced allograft function and chronic rejection [13, 14]. However, whether blockade of IL-21 signaling can effectively prevent chronic immune responses particularly in transplant recipients has not been investigated [8].

Indeed, T cell responses against transplanted organs are always persistent. Thus, we hypothesized that IL-21 is needed for the development of a persistent immune response to cardiac allografts and therefore, its blockade can prevent the development of chronic CAV. Indeed, our results show for the first time that inhibition of IL-21 signaling using either genetic or therapeutic approaches can protect mice from development of CAV.

## Materials and methods

### Mice

Wild-type C57BL/6J (WT), Wild-type BALB/cJ (BALB/c), B6.129S-Batf^tm1.1Kmm/J^ (BATF-/-) and B6(C)-H2-Ab1^bm12^/KhEgJ (B6^bm12^) mice were purchased from the Jackson Laboratory (Bar Harbor, ME, USA). Mutant B6.129S-*Il21*^*tm1Lex*^/Mmcd (IL-21-/-) mice were purchased from the Mutant Mouse Resource and Research Centers (MMRRC) supply at the University of California, Davis. All animals were maintained at the University of Toledo Health Science Campus specific pathogen-free facility. Animal work was performed in accordance with the Guide for the Care and Use of Laboratory Animals of the National Research Council. Protocols for this work were approved by the University of Toledo Institutional Animal Care and Use Committee.

### Skin and heart transplantation

Skin transplants were performed as previously described[15] in WT and IL-21-/- recipient mice from complete MHC mismatched BALB/c donor mice. After confirming acute rejection (within 12 days), these skin transplant recipients were now donor-sensitized and could be used in a model of accelerated-acute rejection of BALB/c heart grafts.

For the chronic rejection model, WT, IL-21-/-, and BATF-/- recipient mice received low immunogenicity heart transplants from B6^bm12^ donor mice. Heart transplants were performed as previously described[16]. Briefly, the heart was excised from the donor, and all vessels to and from the heart were tied off with surgical sutures in 10–0 silk except for the ascending aorta and the pulmonary artery. The ascending aorta of the graft was sutured in to the abdominal aorta of the recipient while the pulmonary artery of the graft was sutured in to the inferior vena cava of the recipient[16].

### Graft survival and function

Survival and function of the heart grafts was monitored by periodic palpation and graded on a continuous scale from zero to four in a blinded fashion, as described by Corry *et al*[17]. Briefly, the location of the graft was palpated, and the impulse was scored by its intensity. A sharp decline in the intensity correlates with the first signs of rejection, while heart graft loss was defined as the point at which the impulse was no longer detectable. Survival of the skin grafts was assessed by daily monitoring for signs of acute rejection such as skin contraction or scabbing[15]. Skin graft loss was defined as the point at which ≥80% of the graft was necrotic[18].

### Fusion protein dosing regimen

A group of WT recipient mice were administered 200 μg (10 mg/kg) of anti-IL-21 receptor fusion protein (IL21R.Fc), or isotype control IgG2 antibody, by i.p. injection on the day of transplantation (post-operative day 0, POD 0) and re-injected every other day until POD 30 (total: 15 injections).

### Histology and cellular analysis

Heart transplants were excised from recipient mice at respective time points. The apex and superior portions of the heart were discarded, and the remaining portion was cut into three equal transverse sections. The center section was placed in acidified methanol for ≥24 hours before being de-hydrated, drained, and fixed in paraffin wax. One of the two remaining sections was fixed with optimal cutting temperature (O.C.T.) medium.

Slides for histological analysis were cut to 4 μm thickness. For evaluation of arterial narrowing, slides were stained for elastin (Verhoeff-van Gieson) and cardiac vessels were imaged using an Olympus FSX100 microscope. Analysis was performed using ImageJ1 (Version 1.51n). Tracings were made of the internal elastic membrane and the lumen, and the amount of vessel occlusion was calculated as $\%\;occlusion=\frac{\left(internal\;elastic\;lamina\;area)-(lumen\;area\right)}{\left(internal\;elastic\;lamina\;area\right)}*100.$ For evaluation of cellular infiltrates, slides were stained with immunological markers for the identification of T-cells (CD3), B-cells (B220), and macrophages (MAC2).

### ELISpot

Cellular production of IL-2 and interferon-γ (IFN-γ) was assessed using an Enzyme-linked immunospot (ELISpot) method described previously by Drs. Valujskikh and Heeger[19]. Briefly, plates were pre-coated with either 4 μg/mL purified anti-mouse IFN-γ or 1 μg/mL purified anti-mouse IL-2 (BD-PharMingen). The next morning, splenocytes from transplant recipients were co-cultured with irradiated antigen presenting cells from donor mice in a 1:1 ratio. Following 24 hours of incubation, cells were washed away, and plates were stained with secondary (Biotin rat anti-mouse IFN-γ or IL-2, 200 or 400 ng/mL respectively, BD-PharMingen), and then tertiary antibodies (Alkaline phosphatase-conjugated anti-biotin) followed by 1-Step NBT/BCIP substrate solution (ThermoFisher Scientific). Developed colorimetric spots were counted using an ImmunoSpot® plate reader (CTL Technologies, Cleveland, OH).

### Flow cytometry reagents and methods

Fluorescence conjugated anti-mouse CD4, CD8, and CD19 mAbs were purchased from BD Biosciences (San Jose, CA) or eBioscience (San Diego, CA). Single cell suspensions from spleens and lymph nodes of heart graft bearing mice were stained with respective fluorescence conjugated antibodies in 2% FBS in PBS. Data were acquired on BD FACS Calibur flow cytometer (Beckton Dickinson) and analyzed using FlowJo and WinMdi software.

### Statistical analyses

Statistical analysis of graft survival curves was performed using the log-rank test. Statistical analysis of vessel occlusions was performed using an unpaired, two-tailed Student's t-test to determine statistical significance at the levels indicated. All tests were performed with GraphPad Prism version 7.04 (GraphPad Software, Inc.; San Diego, CA).

## Results

### IL-21 is not required for acute and accelerated allograft rejection

**Fig 1:**
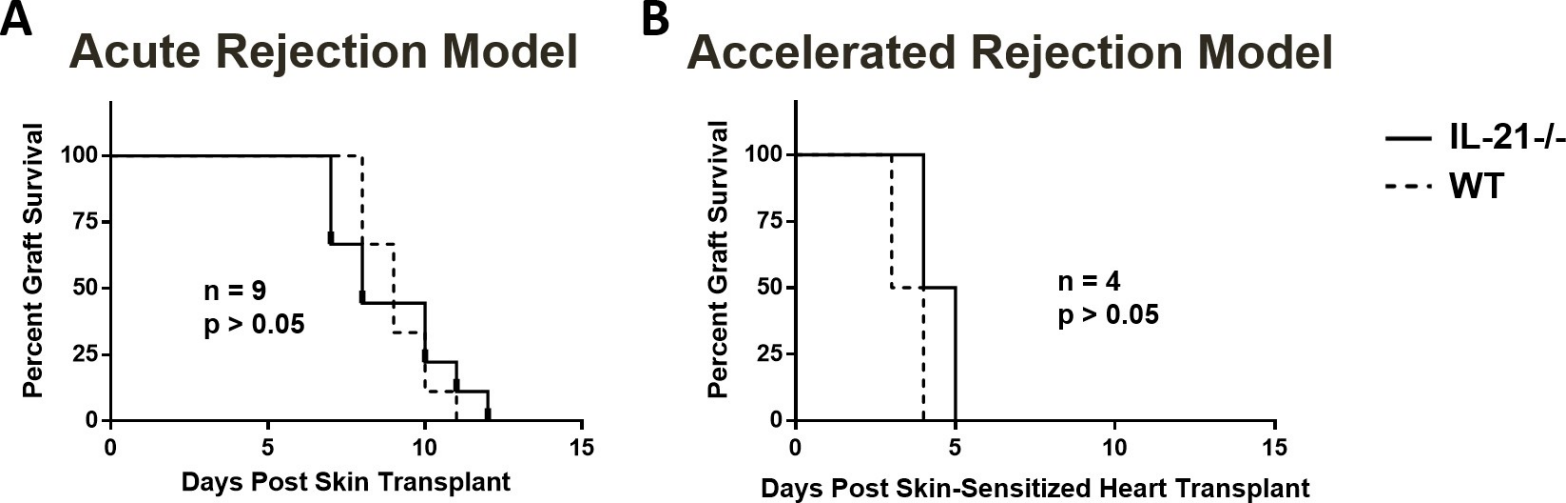
Graft survival in acute and accelerated rejection models. Graft survival over time in WT and IL-21-/- recipient mice of skin transplants from BALB/c donors (A) and BALB/c heart transplants after pre-sensitization with BALB/c skin transplants (B). Significance was calculated by Mantel-Cox test.

Given the results indicating that IL-21 is a chronic phase cytokine[11], we tested the hypothesis that IL-21 production is needed in a chronic but not in acute or accelerated allograft rejection using IL-21 knock-out mice. These mice exhibit normal development of T cells but show reduced numbers of germinal centers [20]. Indeed, an acute rejection model showed no difference in the survival of BALB/c skin transplants grafted to WT or IL-21-/- mice (Fig 1A). Similarly, an accelerated rejection model showed no difference in the survival of BALB/c heart transplants grafted to WT or IL-21-/- mice after pre-sensitization with BALB/c skin allografts (Fig 1B). These results confirmed that IL-21 deficiency in mice did not affect their ability to reject skin and heart allografts in an acute or accelerated fashion.

### IL-21 is required for chronic cardiac allograft vasculopathy (CAV)

**Fig 2:**
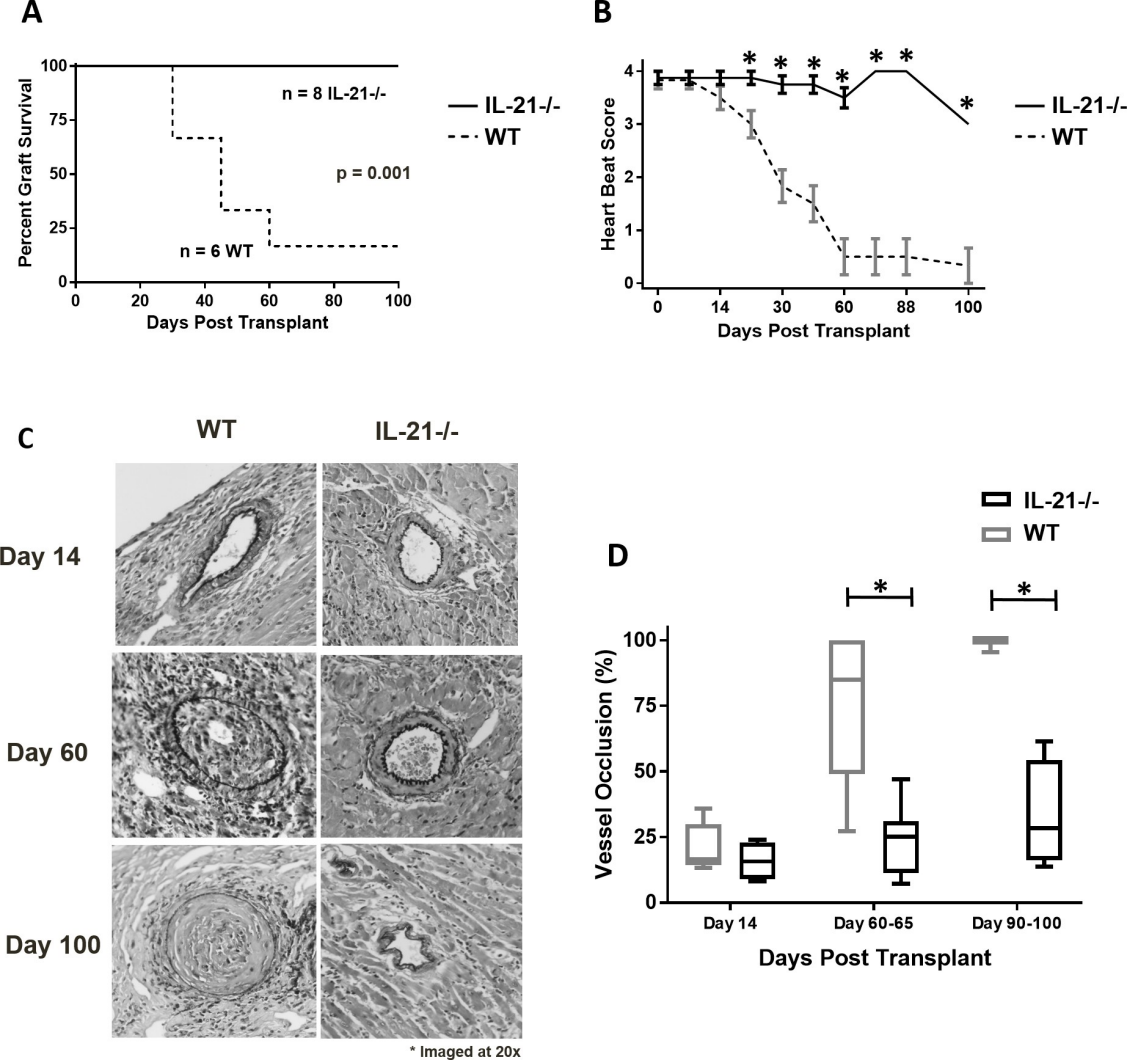
Heart transplant graft evaluation in IL-21-/- mice in a chronic rejection model. Graft survival (A), heart beat scoring (B), and vessel occlusion (C and D) over time in WT and IL-21-/- recipient mice of heart transplants from bm12 mice. Significance approximated by Mantel-Cox test (A), 2-way ANOVA with Bonferroni post-tests (B), and unpaired Student’s t-test (D) * indicates p < 0.05. C) Grafts were recovered on the indicated day, cut, and stained for elastin. D) Percent occlusion was determined by tracing the internal elastic lamina and lumen of each vessel and calculated by $\%\;occlusion=\frac{\left[Area\;of\;internal\;lamina]-[Area\;of\;lumen\right]}{\left[Area\;of\;internal\;lamina\right]}*100$. The number of mice and vessels were as follows: Day 14 (2 mice per group, 9–10 vessels); Day 35–45 (3–4 mice per group, 11–17 vessels); Day 60–65 (6–7 mice per group, 13–27 vessels); Day 90–100 (2 mice per group, 6–8 vessels).

To test whether IL-21 is necessary for the development of CAV, we used a transplant model established to examine chronic allograft rejection [21, 22]: hearts from B6^bm12^ mice, which have three single nucleotide point mutations in the β1 chain of class II H-2/IA MHC, were transplanted in WT or IL-21-/- recipients. Whereas hearts transplanted into WT recipients displayed an anticipated delayed graft rejection [21, 22], demonstrated by graft failure in some mice as early as post-operative day 30 (Fig 2A) and deteriorating heartbeat rates between days 30 and 100 post-grafting (Fig 2B), B6^bm12^ hearts transplanted into IL-21-/- mice survived for more than 100 days post-transplant (Fig 2A) with sustained excellent heartbeat rates (Fig 2B).

The histological analysis of B6^bm12^ hearts transplanted to WT recipients showed characteristic changes of progressive CAV: vessels without changes at day 14 (Fig 2C; upper left) were partially occluded by day 60–65 (Fig 2C; middle left) and completely occluded at day 90–100 (Fig 2C; lower left). In contrast, vessel occlusion was either absent or significantly reduced at all time points (Fig 2C; middle right and 2D; lower right) in IL-21-/- recipients. The results of heart graft function confirmed by histological changes indicate that IL-21 regulates the development of CAV.

### IL-21 regulates interstitial inflammation during CAV development

**Fig 3:**
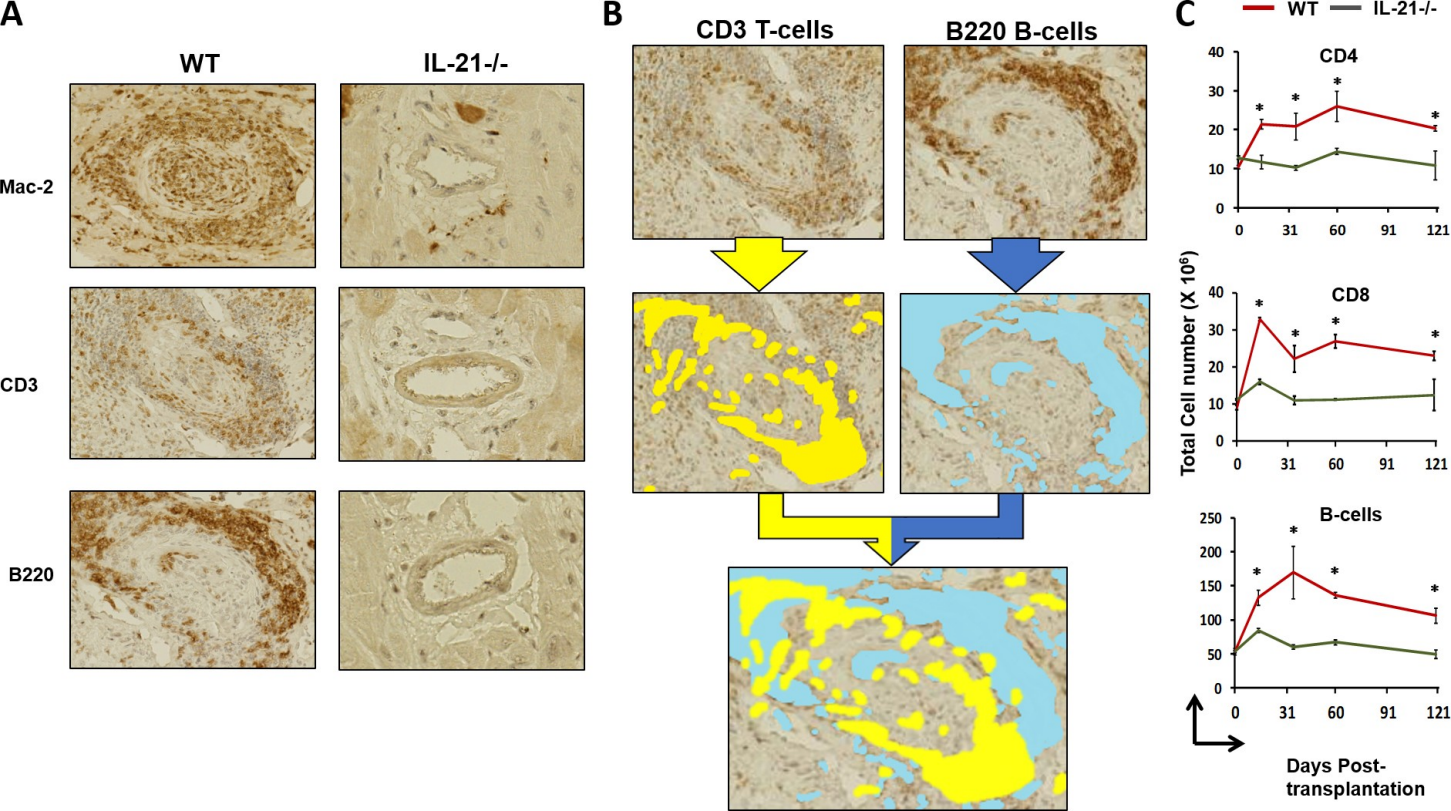
Immune cell infiltration of grafts in IL-21-/- mice in a chronic rejection model. A) Representative images of IHC stained vessels in grafts of heart transplants from bm12 mice in WT or IL-21-/- recipient mice (n = 3 mice/group). Stains were as follows: MAC2, macrophages; CD3, T-cells; B220, B-cells. B) Organization of CD3 stained T-cells and B220 stained B-cells near vessel with possibility of tertiary lymphoid organ like development. C) Accumulation of immune cells detected by flow cytometry of spleen homogenates. Significance approximated by unpaired Student’s t-test, * indicates p < 0.05.

Next, we examined the impact of IL-21 deficiency on leukocyte infiltration in the grafts. At day 100 post-grafting of B6^bm12^ hearts in WT recipients, occlusion (Fig 2C; left panels and 2D) correlated with intensive infiltration by relatively organized clusters of macrophages (Mac-2), T-cells (CD3) and B-cells (B220) (Fig 3A; left panels). Both T and B-cells in the WT recipients were abundantly present in areas around the occluded arteries (Fig 3B; upper and middle panels). Interestingly, while T-cells were generally more proximal to the arteries, some T-cell areas co-localized with nearby B-cells (Fig 3B; lower panel). In contrast, B6^bm12^ hearts in IL-21-/- recipients lacked infiltration of macrophages, T or B-cells or any indication of their presence around the arteries (Fig 3A; right panels). This manifestation of infiltration in WT recipients also correlated with increased numbers of CD4^+^ and CD8^+^ T-cells as well as B-cells in the spleen (Fig 3C). These results suggest that development of CAV may depend on IL-21 promoting local and systemic inflammation driven by T and B-cells.

### Generation of IL-2- and IFN-γ-producing T-cells during CAV development

**Fig 4:**
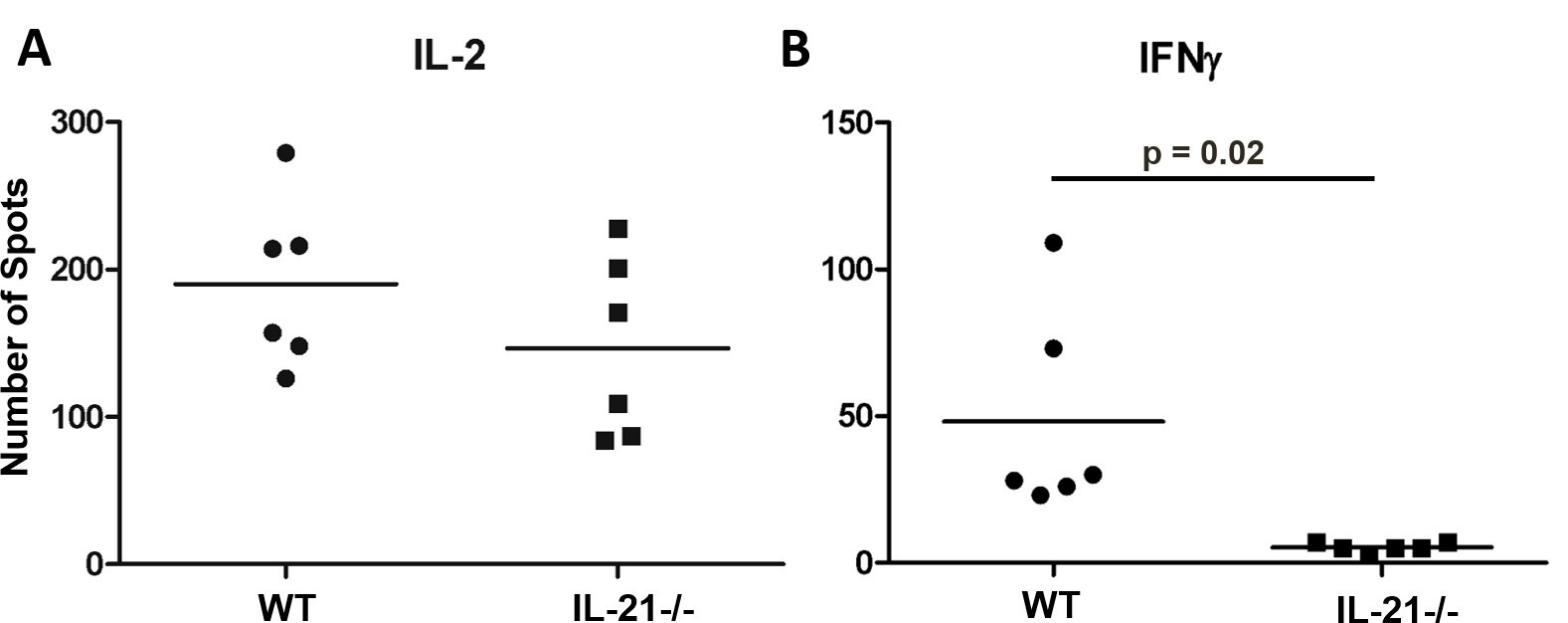
Cytokine production in response to donor antigen. Cytokines produced during co-culture of recipient immune cells from WT or IL-21-/- mice with irradiated bm12 derived antigen presenting cells in a 1:1 ratio. Measured by ELISpot assay in technical triplicates of n = 2 mice. P value from unpaired Student’s t-test.

To test if IL-21 affected the ability of T-cells to produce effector cytokines, splenocytes from recipient mice at 100 days post-grafting were stimulated with irradiated donor cells in an ELISpot assay and analyzed for their ability to produce IL-2 and IFN-γ. Both WT and IL-21-/- recipients presented similar numbers of IL-2 producing cells (Fig 4A), indicating that chronic immune response may not rely on IL-2 production. In contrast, only WT recipients of B6^bm12^ hearts showed significant numbers of IFN-γ-producing cells in comparison to their IL-21 deficient counterparts, suggesting that IL-21 regulated the generation of IFN-γ-producing T-cells (Fig 4B). Therefore, during chronic immune responses, WT mice enhanced IFN-γ production which may contribute to CAV development in B6^bm12^ hearts in an IL-21-dependent fashion.

### BATF transcription factor is required for CAV development

**Fig 5:**
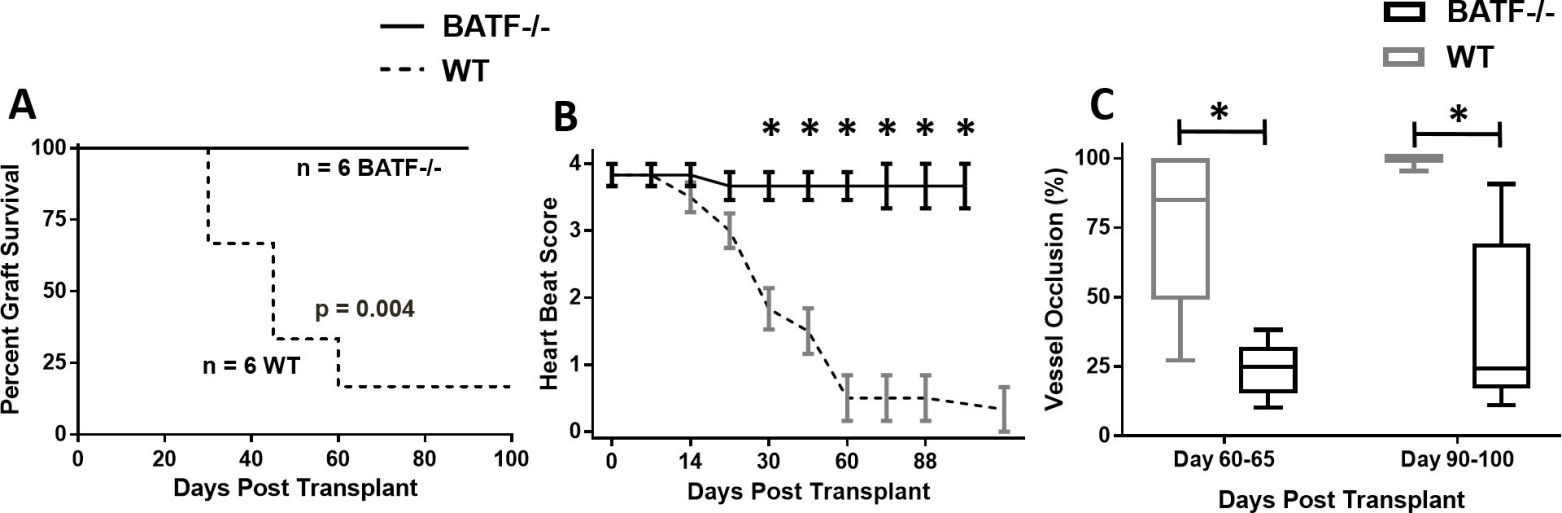
Heart transplant graft evaluation in BATF-/- mice in a chronic rejection model. Graft survival (A), heartbeat scoring (B), and vessel occlusion (C) over time in WT and BATF-/- recipient mice of heart transplants from bm12 mice. Significance approximated by Mantel-Cox test (A), 2-way ANOVA with Bonferroni post-tests (B), and unpaired Student’s t-test (C) * indicates p < 0.05. C) Grafts were recovered on the indicated day, cut, and stained for elastin. Percent occlusion was determined by tracing the internal elastic lamina and lumen of each vessel and calculated by $\%\;occlusion=\frac{\left[Area\;of\;internal\;lamina]-[Area\;of\;lumen\right]}{\left[Area\;of\;internal\;lamina\right]}*100$. The number of mice and vessels were as follows: Day 60–65 (7 WT and 3 BATF-/- mice per group, 16–27 vessels) and Day 90–100 (2–3 mice per group, 8–14 vessels).

To further validate the role of IL-21 signaling in CAV, we evaluated the contribution of the basic leucine zipper transcription factor, ATF-like (BATF) as it regulates IL-21-dependent immune responses[23]. As expected, B6^bm12^ heart allografts in BATF-/- mice had excellent survival (>100 days; Fig 5A) and maintained perfect heartbeat rates (Fig 5B) with absent or significantly reduced CAV at days 60–65 and 90–100 post-transplant compared to allografts in WT recipients (Fig 5C). These results confirm that IL-21 signaling through the transcription factor BATF participates in chronic heart allograft rejection.

### IL-21R.Fc fusion protein prevents CAV development in heart allografts

**Fig 6:**
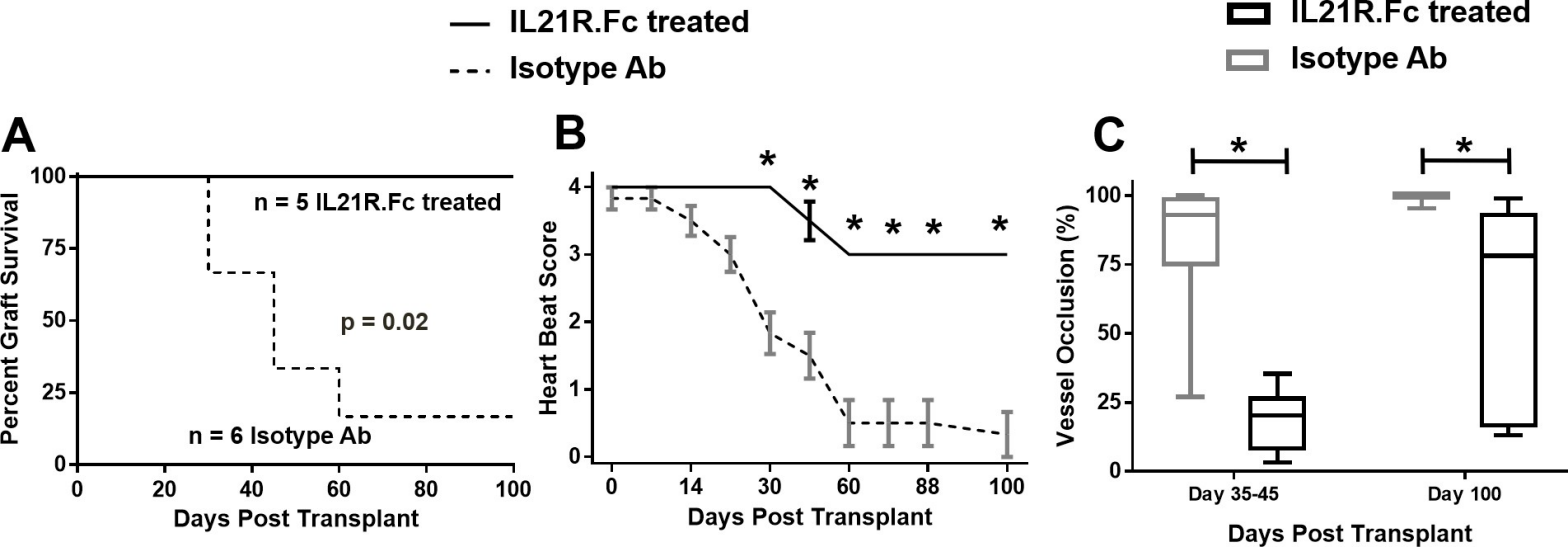
Heart transplant graft evaluation in IL21R.Fc treated WT mice in a chronic rejection model. Graft survival (A), heart beat scoring (B), and vessel occlusion (C) over time in Isotype Ab or IL21R.Fc treated WT recipients of heart transplants from bm12 mice. Significance approximated by Mantel-Cox test (A), 2-way ANOVA with Bonferroni post-tests (B), and unpaired Student’s t-test (C) * indicates p < 0.05. C) Grafts were recovered on the indicated day, cut, and stained for elastin. Percent occlusion was determined by tracing the internal elastic lamina and lumen of each vessel and calculated by $\%\;occlusion=\frac{\left[Area\;of\;internal\;lamina]-[Area\;of\;lumen\right]}{\left[Area\;of\;internal\;lamina\right]}*100$. The number of mice and vessels were as follows: Day 35–45 (3 mice per group, 11–21 vessels) and Day 90–100 (2 mice per group, 8–13 vessels).

We next examined the therapeutic potential of IL-21 blockade using a well-characterized IL-21 receptor fusion protein (IL-21R.Fc)[24]. While allografts in isotype antibody treated WT recipients were rejected between days 30 and 100 post-grafting, all allografts in IL-21R.Fc-treated WT recipients survived more than 100 days (Fig 6A). Additionally, allografts in IL-21 R.Fc-treated WT recipients maintained significantly higher heartbeat scores than those in the control group (Fig 6B). As expected, functional changes in grafts of the control group correlated with a severe CAV pathology at days 35–45 and especially at days 90–100. In contrast, allografts from the IL-21 R.Fc-treated WT recipients presented significantly reduced CAV progression (Fig 6C). These results suggest the therapeutic potential of targeting IL-21 to prevent CAV development and related chronic rejection.

## Discussion

Our results demonstrate that IL-21 is critical for the development of chronic CAV but dispensable in the setting of acute and accelerated rejection of skin and cardiac allografts. This suggests that the availability of T-cell-derived IL-21 is essential for the maintenance of a chronic alloimmune response, conceptually parallel to its role in a chronic viral infection[11]. Moreover, blockade of IL-21 signaling using an IL-21R-Fc fusion protein completely prevented CAV development in wild-type mice, signifying the therapeutic potential of this signaling pathway in chronic allograft rejection.

Heart allografts with chronic rejection lesions have tertiary lymphoid organ (TLO) like structures described as ectopic germinal centers surrounding small arteries with CD23^+^ follicular dendritic cells, B-cells and T-cells, organized in close proximity to an occluded artery[25]. Indeed, IL21 producing Tfh cells are known to be associated with formation of tertiary lymphoid structures in human breast cancer patients in a CXCL13-dependent fashion [26]. Because these TLO-like structures were absent in B6^bm12^ heart allografts in IL-21-/-, BATF-/-, and WT recipients treated with IL-21R.Fc, we conclude that CAV with TLOs develop in the presence of inflammatory cells requiring IL-21 for their survival and effector function. Thus, therapy with IL-21R.Fc fusion protein may prevent these events and have an important clinical application to block CAV.

While studies have shown that both IL-2 and IL-21 are extensively produced during acute allograft rejection[27], our results suggest that lack of IL-21 did not affect the rate of acute or accelerated rejection. This is not surprising as deficiency of IL-21 did not impact the ability of CD4 and CD8 T-cells to resolve acute lymphocytic choriomeningitis virus (LCMV) infections[11]. In contrast, chronic immune responses are dependent on the IL-21/Stat3 signaling axis in Tfh as well as CD8 T-cells[28]. Indeed, our previous study showed that both CD4 and CD8 T-cell survival in the absence of IL-2 signaling was dependent on IL-21 production by CD4 T-cells[29]. Moreover, in our chronic allograft rejection model, IL-21-dependent T-cells produced IFN-γ, which is consistent with the finding that IL-21 sustains T-bet transcription factor in Th1 cells and cytotoxic T cells promoting IFN-γ production[30, 31]. Thus, our results re-emphasize the critical role of IL-21 signaling in mediating chronic immune responses, and for the first time demonstrate it in the context of CAV and chronic allograft rejection.

Transcription factor, BATF is necessary for IL-21 production by Tfh and Th17 cells. In BATF-/- mice, Tfh and Th17 cells fail to mature because BATF controls the expression of their transcription factors Bcl-6 and c-Maf[32, 33]. Our study showed that BATF deficiency reproduced the results of IL-21-/- with improved heart graft survivals and absence of CAV histological changes. Thus, chronic rejection of allogeneic hearts may be mediated by IL-21-dependent Tfh and Th17 cells as BATF needs to be present for the selective development of these functional subsets. There is evidence for Th17 participation in the pathologic remodeling of CAV as B6^bm12^ heart allografts in IL-17-/- recipients had better heartbeat score with reduced occlusion of vessels, similar to our findings[34].

The presence of donor-specific antibodies (DSA) in allograft recipients is known to be an important risk factor for graft loss due to antibody mediated rejection [35]. While the role of DSAs in acute rejection is well-established, emerging evidence also suggests they play a role in chronic antibody mediated rejection [36]. However, published studies have reported that in the B6^bm12^ to B6 MHC-II mismatch chronic rejection model, allo-antibodies were not observed in the heart transplant recipients even though B cells were critical for mediating chronic allograft rejection [37, 38]. Moreover, Zheng et al. demonstrated that B cells actively contribute to CAV in this model by supporting T cell responses through antigen presentation and maintenance of lymphoid structures. Indeed, we observed a striking decrease in B cell infiltration in the B6^bm12^ allografts of IL21-/- mice, which correlated with lack of tertiary lymphoid structures around them. Thus, we believe that B cells and humoral responses may be critical for IL-21 mediated chronic rejection of allografts and we plan to further investigate this aspect in future studies.

In summary, we have documented in three independent models (IL-21-/-, BATF-/-, and IL-21R.Fc therapy) that IL-21 is required for CAV development in heart allografts. In each of these models, lack of IL-21 prevented vessel occlusion. Development of CAV coincided with vessels being surrounded by well-organized TLO-like clusters of macrophages, T and B-cells. In contrast, IL-21 was not required for the development of acute or accelerated rejection. Furthermore, the BATF-deficient model suggests that IL-21-dependent Tfh/Th17 cells may be involved in CAV development during chronic rejection. Most importantly, our study showed that IL-21R.Fc fusion protein therapy inhibited CAV, suggesting a possible clinical application. Hence, we propose that IL-21 is a fundamental player in the development of CAV in heart allografts and a potential therapeutic target.
